# One-Pot Synthesis of Pt Nanobowls Assembled from Ultrafine Nanoparticles for Methanol Oxidation Reaction

**DOI:** 10.3390/nano12193471

**Published:** 2022-10-04

**Authors:** Shoulin Zhang, Pu Wang, Yaoshun Chen, Wenqing Yao, Zhijuan Li, Yawen Tang

**Affiliations:** 1 Jiangsu Key Laboratory of New Power Batteries, Jiangsu Collaborative Innovation Centre of Biomedical Functional Materials, School of Chemistry and Materials Science, Nanjing Normal University, Nanjing 210023, China; 2 School of Environmental Science, Nanjing Xiaozhuang University, Nanjing 211171, China

**Keywords:** Pt nanobowls, ultrafine particles, N, N′-methylenebisacrylamide, methanol oxidation reaction

## Abstract

Simultaneously engineering a bowl-like and ultrafine nano-size structure offers an attractive route to not only increase the utilization efficiency of noble metals, the specific surface areas and the availability of active sites, but also boost the structural robustness and long-term stability. However, a great challenge remains in terms of the methods of synthesis. Herein, we report a facile one-pot hydrothermal method for the preparation of hollow porous Pt nanobowls (NBs) assembled from ultrafine particles. N,N′-methylenebisacrylamide (MBAA) acts as a structure-directing agent that forms a self-template with Pt ions and drives the nucleation and assembly of Pt metals, resulting in the fabrication of Pt NBs from ultrafine particles. By virtue of their unique structure and morphology, the optimized Pt NBs exhibited enhanced electrocatalytic methanol oxidation reaction (MOR) activity with 3.1-fold greater mass activity and 2.6-fold greater specific activities compared with those of commercial Pt black catalysts, as well as excellent stability and anti-poisoning ability.

## 1. Introduction

Facing a series of problems caused by the consumption of fossil fuels and environmental pollution, there is an urgent need to develop sustainable and clean energy conversion devices as substitutes for conventional internal combustion engines [1,2,3,4]. In this context, direct methanol fuel cells (DMFCs) have rapidly attracted increasing attention among the various renewable energy devices due to their high energy conversion efficiency, high power density, ready availability for transportation and storage, and zero carbon footprint [5,6,7,8]. However, the sluggish kinetics of anodic MORs has severely impeded the progress and application of DMFCs [7,9,10]. Hitherto, Pt nanomaterials are still regarded as the most efficient and extensively used MOR catalysts due to their extraordinary affinity for methanol molecules and minimal activation barriers [11,12,13,14,15]. Although many merits of MORs have been recognized, the scarcity, insufficient durability and CO poisoning of commercial Pt electrocatalysts are still major challenges affecting the future development of DMFCs [16,17,18]. Therefore, it remains desirable to develop high-efficiency Pt electrocatalysts with minimal Pt content as well as promising activity and durability towards MORs [19,20,21].

One effective strategy is to tune the size and morphology of Pt catalysts, because the electrochemical performance of catalysts is also highly dependent on these in addition to the components [22,23,24]. As shown by many reported cases, the size effect of noble metals plays a key role in catalyzing a series of important chemical reactions [25,26,27,28]. Notably, reducing the particle size can not only maximize the surface exposure and improve the utilization efficiency of noble metals’ atoms, but can also simultaneously affect the electronic structure of the active metals [29,30,31,32,33,34,35]. Fan et al., for example, studied the catalytic activity of gold nanoparticles with different sizes. The experimental results showed that the catalytic performance decreased with an increase in the nanoparticles’ size [36]. Yang et al. investigated the effect of the size of PdCu nanocatalysts on the ORR activity. The ORR mass activity of PdCu nanoparticles increased from 0.135 A·mg^−1^ to 0.307 A·mg^−1^ as the size decreased from 9.3 nm to 5.8 nm. Density functional theory (DFT) studies have revealed that the edges and corners of metal nanoparticles increase with decreasing size, leading to enhanced ORR catalytic activity [37]. However, controlling the size of ultrafine Pt nanoparticles (under 5 nm) remains a challenge due to their high specific surface energy, which is prone to aggregation and migration during synthesis and electrocatalysis, thus greatly diminishing their catalytic performance [38,39]. The common strategies of integrating the supports to control the nanoparticle size suffer from multiple synthesis steps, non-uniform particle sizes and reduced exposure of the catalyst’s active sites [40,41]. To overcome these difficulties, the uniform assembly of ultrafine nanoparticles into a hollow and porous bowl-like structure is a powerful and unusual strategy, which not only maintains robust stability to prevent the aggregation and migration of ultrafine nanoparticles without introducing supports, but also inherits the high catalytic activity of ultrafine nanoparticles and ensures the maximum retention of active sites [42,43,44,45,46]. Furthermore, the unique hollow bowls are beneficial to provide more open pores and fewer diffusion blockages, resulting in the rapid and efficient entry of reactants and electrolytes into the void space, accelerating the reaction kinetics [47,48,49,50]. However, similar to ultrafine nanoparticles, the synthesis of hollow bowl-like structures still faces great challenges. The template method and galvanic displacement reactions are widely used to synthesize hollow porous structures, which often encounter setbacks such as complicated operation and multiple steps [51,52,53]. Therefore, it is of great significance to develop a simple and effective synthesis strategy that can integrate the merits of ultrafine nanoparticles and hollow porous nanobowls.

Inspired by the approaches above, herein, we developed a simple and ingenious one-pot hydrothermal approach to synthesize Pt NBs assembled from ultrafine nanoparticles by utilizing MBAA. MBAA possesses two amide groups (Figure S1) that can efficiently complex Pt ions, inducing the reduction of Pt ions and the formation of bowl-like structures. Such unique hollow and porous nanobowls endow the catalysts with large surface areas, rich active sites and rapid permeability. As electrocatalysts for the MOR, the as-prepared Pt NBs exhibited superior specific activity (2.8 mA cm^−2^) and mass activity (507.8 mA mg^−1^), as well as enhanced stability and CO_ads_ poisoning tolerance compared with commercial Pt black catalysts.

## 2. Materials and Methods

### 2.1. Reagents and Chemicals

MBAA was purchased from Alfa Aesar Co., Ltd., Heysham, UK. Hexachloroplatinic (IV) acid hexahydrate (H_2_PtCl_6_ · 6H_2_O) was purchased from Shanghai Dibai Biotechnology Co., Ltd, Shanghai, China. Formaldehyde (HCHO) and methanol (CH_3_OH) were purchased from Sinopharm Chemical Reagent Co., Ltd., Shanghai, China. The Pt black catalyst was ordered from Johnson Matthey & Co., Ltd., USA. All reagents were of analytical grade and were used without further purification.

### 2.2. Methods

To synthesize the Pt NBs, 1 mL of a 0.05 M H_2_PtCl_6_ solution and 50 mg MBAA were added into 17.0 mL deionized water with continuous ultrasonic stirring for 1 h at room temperature. Next, 1 mL of a HCHO solution was added into this solution. After ultrasonic treatment for 5 min, the resulting solution was transferred to a 25 mL Teflon-lined stainless steel autoclave and heated at 160 °C for 6 h. After being cooled to room temperature, the final product was collected by centrifugation at 15,000 rpm for 5 min and further purified by washing three times with distilled water and ethanol.

### 2.3. Characterization

Transmission electron microscopy (TEM) and high-resolution TEM (HRTEM) images of the Pt NBs were acquired on Talos G2 F20 and JEOL JEM-2100F instruments, manipulated with an accelerating voltage of 200 kV. Scanning electron microscopy (SEM) studies were performed using a Hitachi S4800 system. XRD analyses were performed on a Model D/max-rC X-ray diffractometer using Kα radiation (λ = 0.15406 nm). XPS measurements were conducted on a Thermo VG Scientific ESCALAB 250 spectrometer with Al Kα radiation. The binding energy was calibrated by means of the C 1s binding energy at 284.6 eV.

### 2.4. Electrochemical Measurements

Electrochemical measurements were performed on a CHI 760E CH analyzer (Shanghai, China, Chenhua Co., Ltd.) with a conventional three-electrode system. The glassy carbon electrode loaded with a catalyst was used as the working electrode, with the graphite rod as the counter-electrode and a saturated calomel electrode (SCE) as the reference electrode for the MOR tests. The catalyst ink was prepared by ultrasonically dispersing a mixture of 2.0 mg of the catalyst and 1 mL of deionized water. Next, a 6 μL drop of the suspension was coated on a glassy carbon electrode with a diameter of 3 mm. After drying, 3 μL of a Nafion solution (5 wt.%) was added onto the surface of the modified electrode and dried again naturally at room temperature. Electrochemical measurements were performed in a 0.5 M N_2_-saturated H_2_SO_4_ solution with or without 1.0 M CH_3_OH at a scan rate of 50 mV s^–1^. Chronoamperometry curves were obtained in a N_2_-saturated 0.5 M H_2_SO_4_ + 1 M CH_3_OH mixed solution for 85,000 s at 0.6 V.

## 3. Results and Discussion

**Figure 1:**
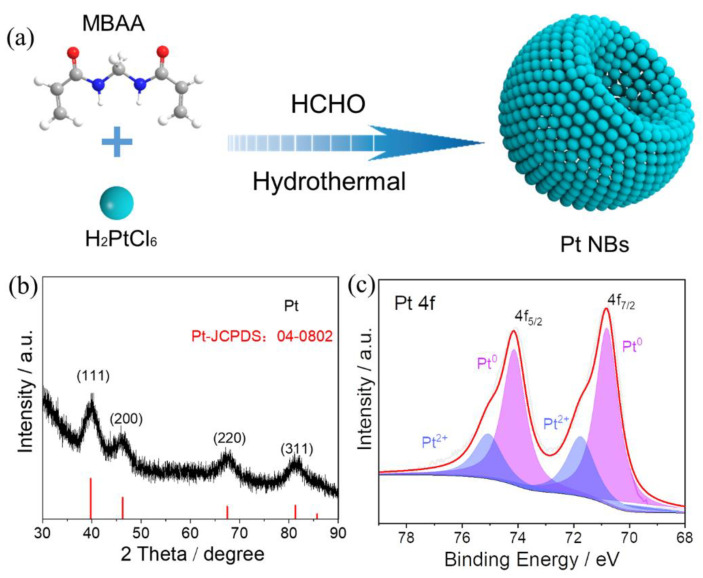
(**a**) Schematic illustration of the procedure of synthesis, (**b**) the XRD pattern and (**c**) the high-resolution Pt 4f XPS spectrum of Pt NBs.

Figure 1a illustrates the one-pot synthesis of Pt NBs. In a typical process, the Pt NBs were successfully constructed with the assistance of MBAA, using H_2_PtCl_6_ as the metal precursor and HCHO as the reducing agent. The crystalline nature of the Pt NBs was confirmed by powder X-ray diffraction (XRD). As obviously shown in Figure 1b, the diffraction peaks located at 39.8°, 46.2°, 67.5° and 81.3° corresponded well to the (111), (200), (220) and (311) facets of the face-centered cubic *(fcc)* phase of Pt (JCPDS No. 04-0802), respectively, indicating the formation of Pt metal. The surface compositions and electronic states of the elements in Pt NBs were investigated by XPS. Figure 1c shows that the deconvoluted Pt 4f spectrum of Pt NBs was composed of Pt^0^ and Pt^2+^ species. The peaks located at 70.8 and 74.1 eV were attributed to Pt 4f_7/2_ and Pt 4f_5/2_ of Pt^0^ species, respectively, whereas the other low peaks at 71.8 and 75.1 eV can be attributed to Pt^2+^ species [54]. Through integration of the peak areas, 72.3% of the Pt elements were reduced to metallic Pt^0^, verifying the effective reduction of Pt^4+^. Compared with that of standard metallic Pt (4f_7/2_: 70.90 eV), the negative shift of about 0.1 eV was probably due to the surface-modifying effect of MBAA on Pt [55]. Moreover, the modification of a small amount of MBAA on Pt was further confirmed by the N 1s spectrum (Figure S2).

**Figure 2:**
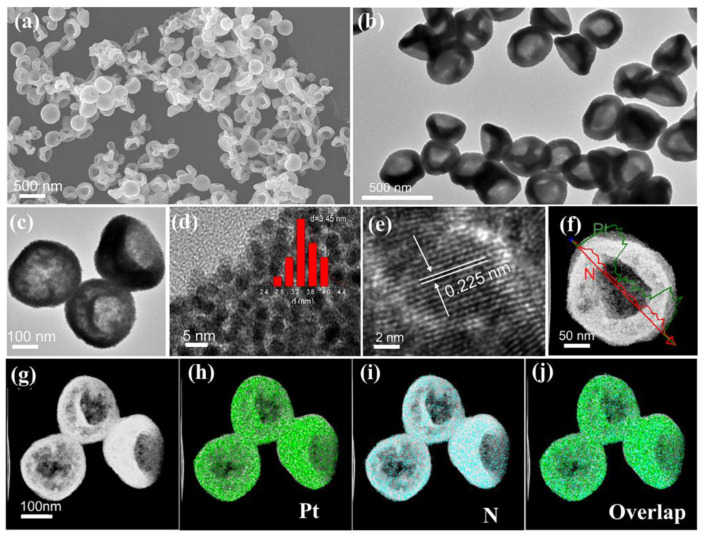
(**a**) SEM image, (**b**,**c**) TEM images, (**d**,**e**) HRTEM images (the insert shows the size distribution histogram), (**f**) STEM image and EDX line scanning profile, and (**g**–**j**) STEM image and the corresponding elemental mapping images of Pt NBs.

The morphology and structure of the resulting Pt NBs were initially characterized by various electron microscopy techniques. As revealed in the SEM images ( Figure 2a and Figure S3), the obtained Pt NBs exhibited a uniformly dispersed bowl-like structure with an almost 100% yield. TEM images at different magnifications further validated the hollow bowl-like nature, with an average diameter of 300 nm (Figure 2b,c). Unlike the traditional single-layer bowl-like structure, the Pt NBs obtained here possessed double-layered shells, similar to a concave hollow spherical structure. Notably, further magnification of the TEM images (Figure 2d and Figure S4) revealed that the obtained concave bowl-like structure was constructed by interconnecting and assembled Pt nanoparticles with a size of about 3.45 nm. Figure 2e presents the HRTEM image of the Pt NBs. The adjacent lattice fringes of the majority of the exposed facets were 0.225 nm, perfectly corresponding to the (111) lattice spacing of fcc Pt. In the enlarged HRTEM image, a large number of lattice defects such as distortions and twin boundaries can clearly be observed on the surface of the Pt NBs, as indicated by the yellow markers (Figure S5). Moreover, the surface of the nanobowl was rough and porous, favorably reducing the mass transfer resistance and improving the reaction kinetics in the electrocatalytic process (red marks in Figure S5) [47,56]. Meanwhile, the hollow and porous bowl-like structure with abundant surface defects could provide large electrocatalytic surface areas and accessible active sites, thus facilitating improvements in the electrocatalytic performance. The clear bowl-like structure was also confirmed by the scanning TEM (STEM) images (Figure 2f,g). As demonstrated in Figure 2f, the dark centers and bright edges can clearly be observed, which are distinct features of nanobowls. The bowl-like structure was further verified by the energy-dispersive X-ray (EDX) line scanning spectra, according to the weaker element signals in the hollow center (inset in Figure 2f). Combined with the EDX element mapping images in Figure 2h–j, the uniform distribution of Pt and a small number of N elements were detected on the surface of the Pt NBs. The modification of a small number of N elements on the surface may induce changes in the electronic structure of Pt, which is beneficial for relieving the adsorption of intermediates, thereby boosting the electrocatalytic reaction performance.

To understand the role of MBAA in adjusting the morphology, comparative experiments with different amounts of MBAA were carried out. In the absence of MBAA (Figure S6a), the products were small agglomerated nanoparticles with no specific morphology. When 5 mg of MBAA was added, nanospheres with a size of about 70 nm were obtained (Figure S6b). As the amount of MBAA increased to 10 mg (Figure S6c), the centers of the nanospheres began to form hollows, accompanied by some by-products. When the amount of MBAA reached 15 mg (Figure S6d), the hollow nanospheres gradually became concave and the by-products decreased significantly. As the amount of MBAA continued to increase to 30 mg (Figure S6e), apple-like structures were formed. The detailed experimental results show that the concave degree of the product gradually increased with the increase in the amount of MBAA. When 50 mg of MBAA was added (Figure S6f), uniform hollow porous nanobowls were finally obtained. These results suggest that MBAA plays an indispensable role in forming the hollow bowl-like morphology of the catalyst. Reasonably, MBAA, as a strong ligand with two special amide groups, can effectively complex with Pt ions. As confirmed by the UV-Vis spectra (Figure S7a), the characteristic absorption peak of the H_2_PtCl_6_ solution at 261 nm disappeared after mixing H_2_PtCl_6_ and MBAA solution, fully confirming the complexation of the two solutions. The complexes thus formed can effectively reduce the reduction rate of Pt ions, thereby reducing the rate of self-nucleation and growth, and regulating the final morphology of the product [57,58]. In addition, FT-IR spectroscopy was performed on the MBAA and the final Pt NBs (Figure S7b). The FT-IR spectra of the MBAA and the final Pt NBs showed that there was a blue shift in the amine group on the surface of the Pt NBs, showing that there was interaction between Pt and the amine group of MBAA. The modification of a small amount of MBAA on Pt is conducive to changing the electronic structure of Pt and easing the adsorption of intermediates, thus improving the electrocatalytic performance [55].

**Figure 3:**
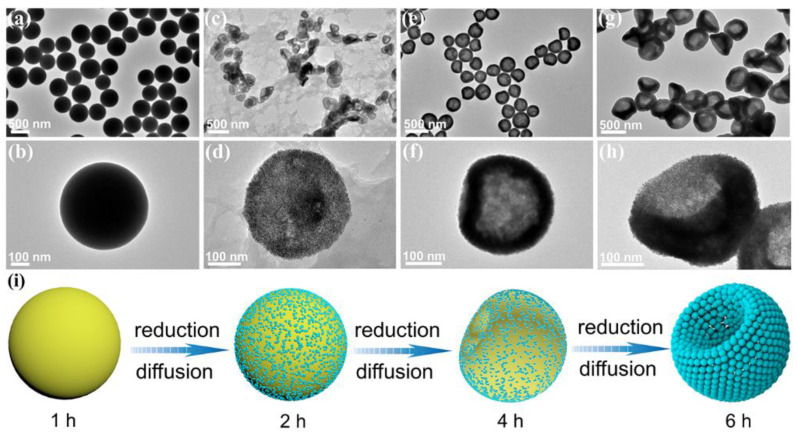
TEM images of Pt NBs at different reaction times: (**a**,**b**) 1 h, (**c**,**d**) 2 h, (**e**,**f**) 4 h and (**g**,**h**) 6 h. (**i**) Schematic illustration of the corresponding products obtained at different reaction times.

To further clarify the morphological evolution of Pt NBs, the intermediates collected at different reaction times were analyzed using the controlled variable method. At the initial reaction time, the products were obtained as yellow powder. Additionally, the corresponding TEM images (Figure 3a,b) showed that the products were uniformly dispersed solid nanospheres with a smooth surface and an average particle size of 500 nm. However, the corresponding XRD pattern (Figure S8a) indicated that there were no characteristic diffraction peaks of Pt. This result revealed that no metal Pt was reduced and the nanospherical products thus obtained were complexes formed by MBAA and Pt ions at high temperature and high pressure. With the prolongation of the reaction time to 2 h, ultrafine nanoparticles gradually grew on the surface of the nanospheres (Figure 3c,d), which were confirmed to be Pt nanoparticles by XRD (Figure S8b). In addition, the nanospheres changed from solid to hollow, with a large amount of by-product, which could be attributed to the continuous diffusion and reduction of Pt ions on the surface of the nanospheres (Figure 3e,f). As the reaction time increased to 4 h, due to the further diffusion of and reduction in platinum ions, the number of complexes as supporting spheres gradually decreased, resulting in the surface depressions of hollow nanospheres. Moreover, with the further diffusion of and complete reduction in Pt ions, the concavity of the hollow nanospheres was further deepened. Finally, hollow and porous bowl-like Pt NBs assembled from ultrafine nanoparticles were obtained when the hydrothermal reaction time reached 6 h (Figure 3g,h). Figure 3i briefly illustrates the structural evolution of the Pt NBs. Unlike the traditional hard template method, using MBAA as the morphology-directing agent to prepare the hollow and porous bowl-like structure not only involves fewer steps and produces less waste, but is also cost-effective. The synthesis involves the complexation of MBAA and Pt ions, the soft template effects of the complex and the diffusion of Pt ions, which will provide new ideas for the formation of bowl-shaped Pt nanomaterials. Notably, bowl-shaped metals or nanomaterials have exhibited great potential in catalysis, energy storage and biomedical applications [52,59].

**Figure 4:**
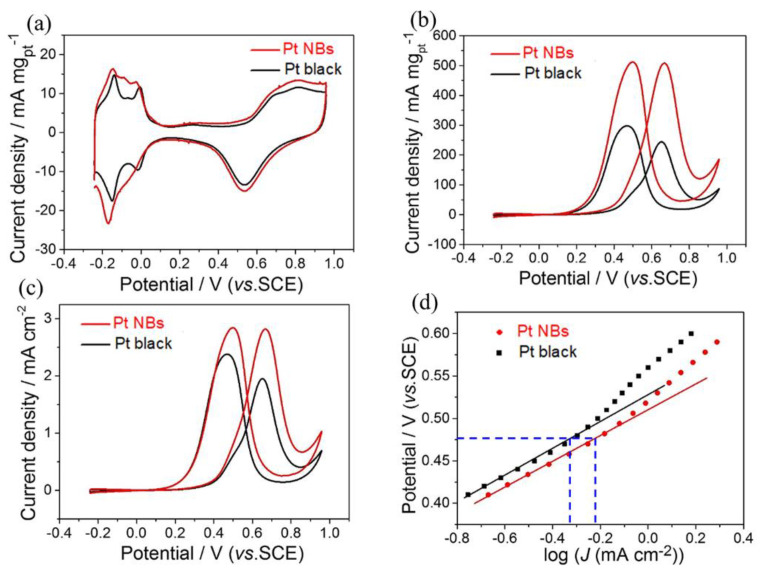
(**a**) CV curves in a 0.5 M H_2_SO_4_ solution at a scan rate of 50 mV s^−1^ and CV curves in a 0.5 M H_2_SO_4_ + 1.0 M CH_3_OH solution at a scan rate of 50 mV s^−1^: (**b**) mass activity, (**c**) specific activity and (**d**) Tafel plots.

Inspired by their unique structural characteristics, the catalytic performance of Pt NBs and commercial Pt black were evaluated for MORs under acidic conditions. Before the electrochemical test, the prepared Pt NB catalyst was immersed in acetic acid for 12 h to remove excess MBAA on the surface. First, cyclic voltammetry (CV) measurements were recorded in a 0.5 M H_2_SO_4_ solution at a scan rate of 50 mV s^−1^. The electrochemically active areas (ECSAs) of the catalysts were calculated by integrating the charge in the hydrogen adsorption–desorption region of the cyclic voltammetry curves thus obtained (Figure 4a). The ECSAs of hollow Pt NBs and commercial Pt black were measured to be 18.2 and 12.5 m^2^ g^−1^, respectively. Due to their larger ECSAs, the Pt NBs were expected to be outstanding MOR catalysts. Therefore, the MOR electrocatalytic activity of the Pt NBs and Pt black catalysts were evaluated by CV curves obtained in a 0.5 M H_2_SO_4_ + 1.0 M CH_3_OH solution. As indicated in Figure 4b, the mass activity of the Pt NB catalyst reached 507.8 mA mg^−1^, with a 2.1-fold improvement compared with the commercial Pt black (242.7 mA mg^−1^). A similar trend also emerged in terms of the specific activity. As shown in Figure 4c, the specific activity of the Pt NBs was calculated to be 2.8 mA cm^−2^, which was 1.4 times higher than that of commercial Pt black (1.9 mA cm^−2^). Figure 4d exhibits the corresponding Tafel curves derived from Figure 4c. The Tafel plots maintained a linear relationship in the low potential region. Additionally, the Pt NBs displayed a higher output current density than the commercial Pt black at the same potential, indicating a faster kinetic rate for methanol electrocatalysis (Figure 4d). Furthermore, the lower Tafel slope of the Pt NBs indicated that there was less coverage of CO or intermediates in the Tafel region compared with commercial Pt black. 

**Figure 5:**
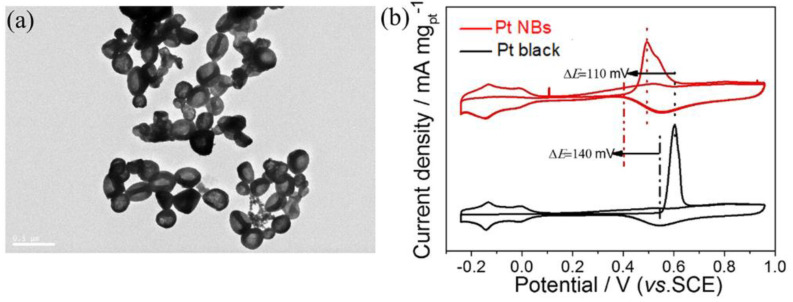
(**a**) TEM images of the Pt NBs after the stability test. (**b**) Pre-absorbed CO-stripping voltammograms in an N_2_-saturated 0.5 M H_2_SO_4_ solution.

Electrocatalytic stability and CO poisoning tolerance are two important indicators for evaluating the performance of the MOR. Chronoamperometry was first used to assess the catalytic stability of Pt NBs and commercial Pt black in an N_2_-saturated 0.5 M H_2_SO_4_ + 1 M CH_3_OH solution at a potential of 0.6 V. As shown in Figure S9, the current density of the Pt NBs was consistently higher than that of the commercial Pt black after 85,000 s, manifesting a more robust MOR stability. In addition, the morphology of the recovered Pt NBs retained the whole bowl-shaped structure without significant changes after the stability test. (Figure 5a). The excellent electrocatalytic stability of the Pt NBs resulted from its hollow porous structure, which effectively inhibited the possible aggregation, dissolution and Ostwald maturation of catalysts. The CO poisoning resistance of Pt NBs towards the MOR was investigated by CO stripping voltammetry (Figure 5b). Compared with those of commercial Pt black, the onset and peak oxidation potential of CO on the Pt NBs were negatively shifted by 140 and 110 mV, respectively, indicating stronger resistance to CO poisoning during the MOR electrocatalysis process.

On the basis of the experimental results and analysis above, we can confirm that Pt NBs are excellent and stable catalysts for anode MOR of DMFCs. This can be attributed to the following aspects: (i) the hollow porous nanobowls and ultrafine nanoparticles can provide large specific surface areas and abundant accessible active sites, which are conducive to the rapid transport of mass and electrons, in favor of improvements in the MOR’s activity; (ii) the ultrafine nano-size and the surface modification of MBAA can tune the electronic structure of Pt and facilitate the desorption of intermediates and toxic species, thereby enhancing the anti-poisoning ability and the reaction kinetics of the catalysts; (iii) the self-supporting property of the hollow bowl-like structure can effectively prevent the agglomeration, leaching and migration of the catalyst, firmly maintaining the whole structure during the electrocatalytic process.

## 4. Conclusions

In summary, we have successfully synthesized hollow porous Pt NBs composed of ultrafine nanoparticles (about 3 nm) via an easy one-pot hydrothermal method of synthesis. A series of experimental studies indicated that MBAA plays a decisive role in tuning the bowl-like structure. The successful integration of the hollow bowl-like structure and ultrafine nanoparticles provided large active areas, accessible active sites and rapid mass transfer channels for the Pt NBs catalyst. In virtue of their favorable morphology and electronic structure, the optimized Pt NBs exhibited remarkably improved electrocatalytic MOR activity, stability and anti-poisoning ability in an acidic medium. This study has presented an efficient method for and furthered our understanding of the formation of bowl-shaped nanomaterials.

## Data Availability

The data presented in this study are available on request from the corresponding author.

## Supplementary Materials

The following supporting information can be downloaded at: https://www.mdpi.com/article/10.3390/nano12193471/s1. Figure S1: Molecular structure of MBAA. Figure S2. High-resolution N 1 s XPS spectrum of Pt NBs. Figure S3: SEM images of Pt NBs at different magnifications. Figure S4: HREM image of Pt NBs. Figure S5: HREM image of Pt NBs. Figure S6: TEM images of Pt NBs with (a) 0 mg, (b) 5 mg, (c) 10 mg, (d) 15 mg, (e) 30 mg and (f) 50 mg of MBAA. Figure S7: (a) UV-Vis spectra of the H_2_PtCl_6_, MBAA and the mixed solution; (b) FT-IR spectra of Pt NBs and MBAA. Figure S8: XRD patterns at (a) 1h and (b) 2 h. Figure S9: Chronoamperometry curves in an N_2_-saturated 0.5 M H_2_SO_4_ + 1 M CH_3_OH solution at 0.6 V.
