# Supplementary material for: One-Pot Synthesis of Pt Nanobowls Assembled from Ultrafine Nanoparticles for Methanol Oxidation Reaction
Source: Nanomaterials (Basel). 2022 Oct 4;12(19):3471. doi: 10.3390/nano12193471 (PMC9565777; doi:10.3390/nano12193471)
Supplement: Supplementary file 1 [file nanomaterials-12-03471-s001.zip › nanomaterials-1914040-SI.pdf]

Article

One-Pot Synthesis of Pt Nanobowls Assembled from Ultrafine Nanoparticles for Methanol Oxidation Reaction

Shoulin Zhang [1,†], Pu Wang [1,†], Yaoshun Chen [1], Wenqing Yao [1], Zhijuan Li [2,*] and Yawen Tang [1,*]

1 Jiangsu Key Laboratory of New Power Batteries, Jiangsu Collaborative Innovation Centre of Biomedical Functional Materials, School of Chemistry and Materials Science, Nanjing Normal University,
Nanjing 210023, China; zhangshoulin@njnu.edu.cn (S.Z.); wangpunnu@163.com (P.W.); chenyaoshunyyds@163.com (Y.C.); ywq18851132255@163.com (W.Y.)

2 School of Environmental Science, Nanjing Xiaozhuang University, Nanjing 211171, China

* Correspondence: zhijuanlibbd@163.com (Z.L.); tangyawen@njnu.edu.cn (Y.T.)

† These authors contributed equally to this work.

Figures and Table

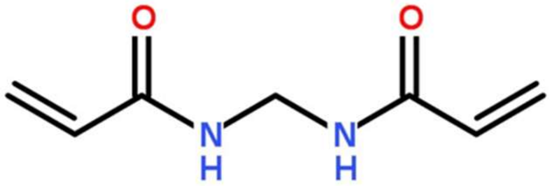

Figure S1. Molecular Structure of MBAA

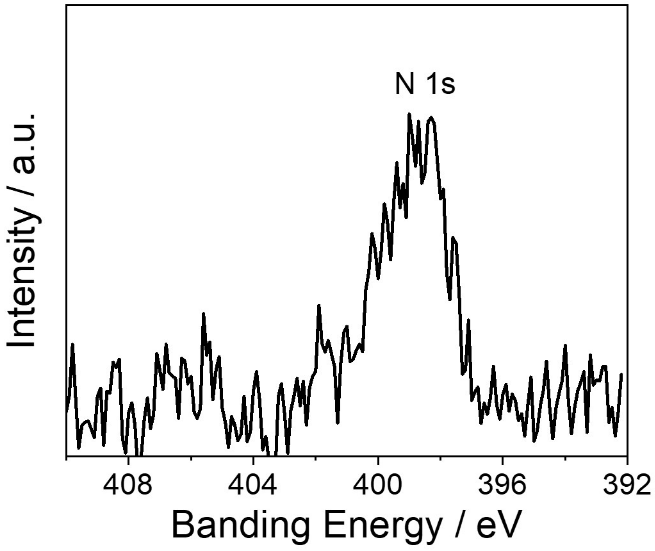

Figure S2. High-resolution N 1s XPS spectrum of Pt NBs.

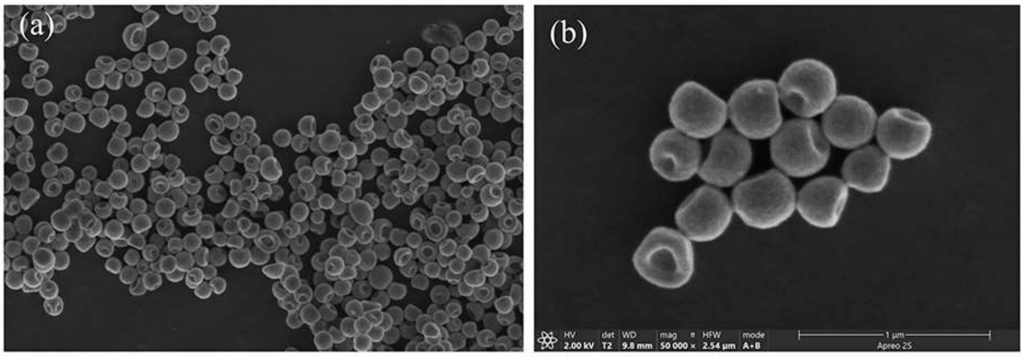

Figure S3. SEM images of Pt NBs at different magnifications

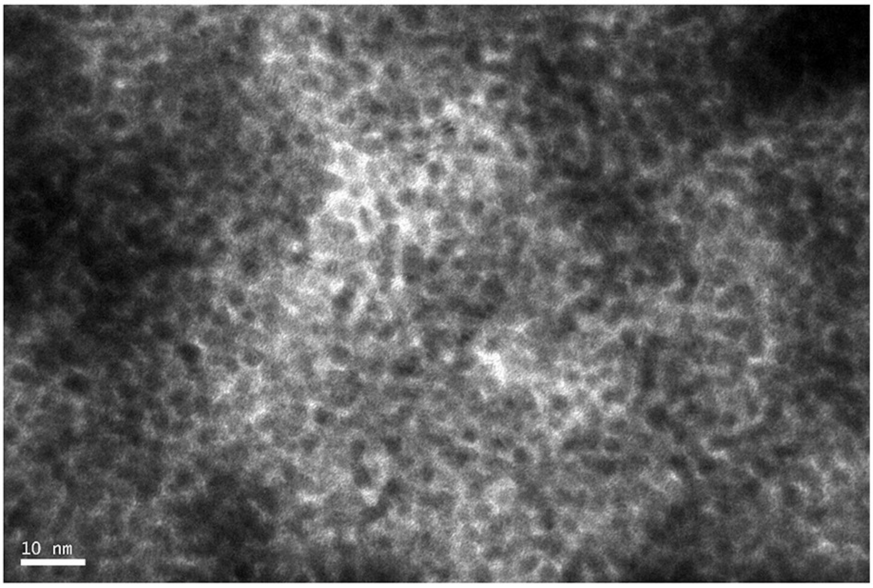

Figure S4. HREM image of Pt NBs.

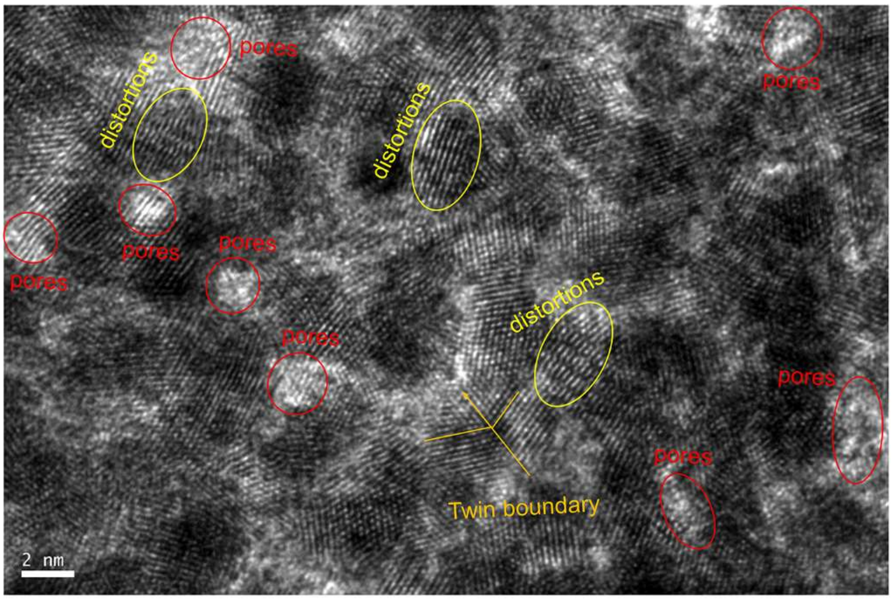

Figure S5. HREM image of Pt NBs

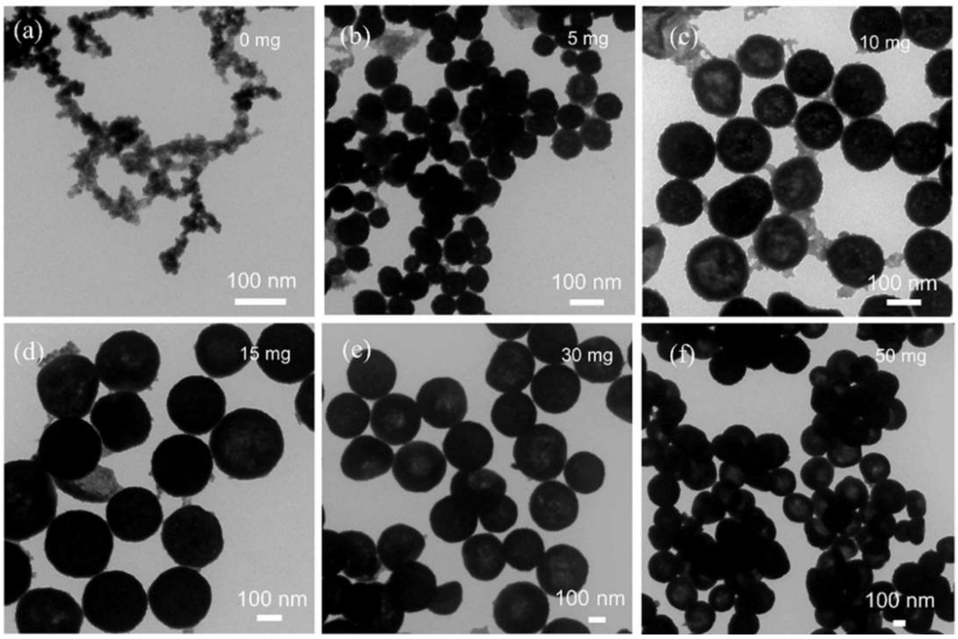

Figure S6. TEM images of Pt NBs with (a) 0 mg, (b) 5 mg, (c) 10 mg, (d) 15 mg, (e) 30 mg and (f) 50 mg of MBAA

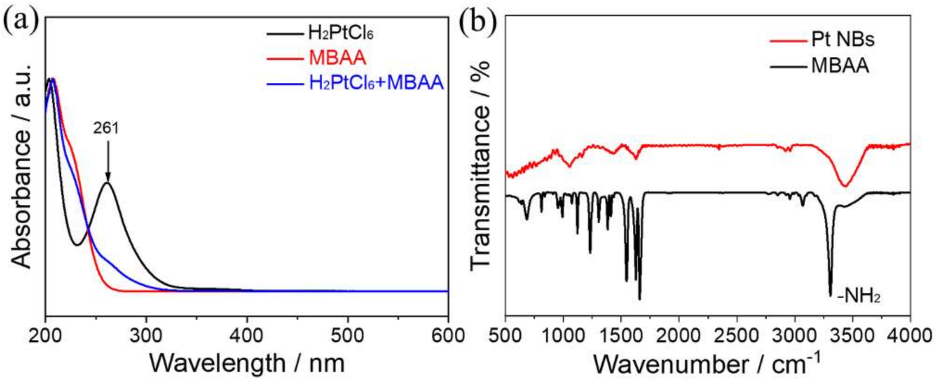

Figure S7. (a) UV-vis spectra of the H_2PtCl_6, MBAA and the mixed solution; (b) FT-IR spectra of Pt NBs and MBAA

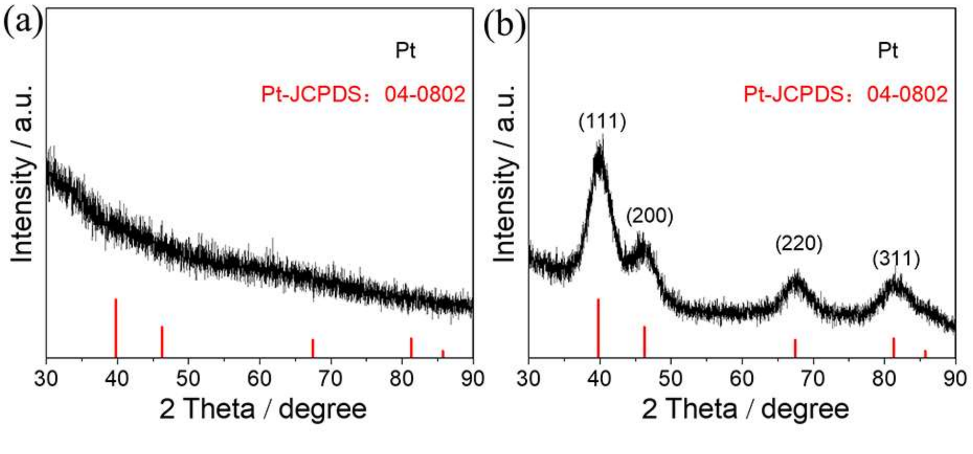

Figure S8. XRD pattern at (a) 1h and (b) 2 h

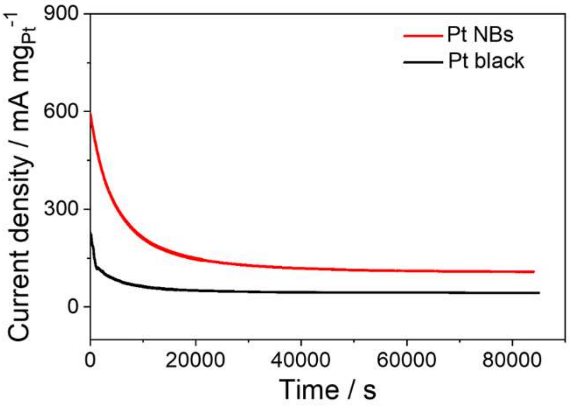

Figure S9. Chronoamperometry curves in a N_2-saturated 0.5 M H_2SO_4 + 1 M CH_3OH solution at 0.6 V
